# Show Me the Money: Successfully Obtaining Grant Funding in Medical Education

**DOI:** 10.5811/westjem.2018.10.41269

**Published:** 2018-11-19

**Authors:** Michael Gottlieb, Sangil Lee, John Burkhardt, Jestin N. Carlson, Andrew M. King, Ambrose H. Wong, Sally A. Santen

**Affiliations:** * Rush University Medical Center, Department of Emergency Medicine, Chicago, Illinois; † University of Iowa Carver College of Medicine, Iowa City, Iowa; ‡ University of Michigan School of Medicine, Department of Emergency Medicine, Ann Arbor, Michigan; § Allegheny Health Network, Department of Emergency Medicine, Erie, Pennsylvania; ¶ The Ohio State University Wexner Medical Center, Department of Emergency Medicine, Columbus, Ohio; || Yale School of Medicine, Department of Emergency Medicine, New Haven, Connecticut; # Virginia Commonwealth University School of Medicine, Department of Emergency Medicine, Richmond, Virginia

## Abstract

Obtaining grant funding is a fundamental component to achieving a successful research career. A successful grant application needs to meet specific mechanistic expectations of reviewers and funders. This paper provides an overview of the importance of grant funding within medical education, followed by a stepwise discussion of strategies for creating a successful grant application for medical education-based proposals. The last section includes a list of available medical education research grants.

## INTRODUCTION

Promotion of faculty in academic institutions is critical and includes service, education, and research agendas. The development of faculty researchers has been a topic of interest and debate. Studies in this arena have primarily focused on leadership practices,^1^ theoretical frameworks,^2^ and barriers to success.^2^,^3^ A recurrent theme in these works is the importance of obtaining significant extramural research support in an increasingly competitive funding environment.^4^ In fact, early career researchers who successfully obtain funding have nearly twice the likelihood of achieving full professorship compared to those without funding, even with similar pre-award backgrounds.^5^

Interestingly, the ability to initially predict successful grant applicants from non-successful applicants based upon their qualifications alone can be difficult, as they often have similar backgrounds and credentials.^6^ However, once funding has been achieved, the likelihood of receiving subsequent grants significantly increases.^7^ This phenomenon, the degree to which the successful become ever more successful, has even been given its own term in the social sciences and is referred to as the Matthew effect.^7^,^8^ While emergency medicine is a young field, funded research is gaining importance and recognition.^9^ Achieving grant funding can be incredibly important to building and gaining momentum in one’s academic career.

Grant funding is particularly important in the field of medical education research, where funding is even more scarce^10^–^12^ and the work itself is often under-recognized.^13^–^15^ Additionally, medical education research is striving to be more rigorous. Experts have suggested that the majority of medical education research is either unfunded or underfunded,^1^,^2^ which may negatively impact the quality of the studies.^3^ The scientific community, including professional organizations, journal editors, and investigators, have all called for a higher caliber of methodological rigor, multi-institutional studies, and clinically-relevant outcomes for medical education research.^3^,^16^,^17^ To achieve these goals we believe that educational researchers must have the tools and information necessary to successfully apply for grant support. We created this document to provide an overview of the grant application process, including mechanics of a grant, key sections and how to apply, as well as to provide a list of potential medical education funding mechanisms. We hope this will help early career investigators obtain medical education funding.

## ANATOMY OF A GRANT APPLICATION

### Overview

Completing a grant application can be a large and arduous task. Grant applicants will often need several experts to formulate their team. The grant development team should include a principal investigator (PI), a primary mentor who is ideally from the same institution, and at least one content expert in the topic of interest. A content expert could be a clinical, translational, educational, or statistical expert either within or outside your institution. It is also important to include a methodological expert early in the process, particularly if the PI does not have significant expertise with the technique. For example, if an investigator is performing a qualitative study, it would be important to include someone who has significant experience with this approach to ensure that the qualitative approach (e.g., phenomenology, grounded theory) and specific methodology is appropriate and rigorous. It is also important to establish strong collaboration with the team, rather than merely listing prominent names without previous collaboration. Therefore, we strongly recommend that the PI start the preliminary work early by assigning specific roles among team members and involving them in the grant process.

While the specific structure of individual grants can vary by sponsoring institution, many grants use similar requirements and formats. The specific format typically includes but is not limited to a project description with references, biosketches of the investigative team, a complete budget with justification of the financial expenditures, a description of the facilities and resources, and letters of support. The following sections of this paper provide a brief summary of each of these commonly included elements within grant applications; however, the reader is advised to spend additional time reviewing the individual funders’ websites and application resources to ensure that all required forms are completed appropriately and included within the final grant application. It is also important to check with your institution’s grant office, which often requires approval to submit a grant. This process can take up to a month, so it is important to start the work early on grant applications.

### Project Description

The project description section serves as the body of the grant and clearly defines the work to be performed. The allotted length varies by the funding mechanism, but often ranges from 6–12 pages. The first page of the application is typically the specific aims page where the project is summarized in two or three paragraphs and the hypotheses and objectives are explicitly defined. Well-designed aims ensure that success is not dependent upon any single outcome and that more than one possible outcome is acceptable. Success of a subsequent aim should not be dependent upon the prior aim. Depending upon the funding mechanism, the specific aims page may or may not be included in the page count for the project description. This section is often considered the most important page in the entire grant application. Significant time should be spent on this page by all members of the grant team to ensure that it clearly and accurately articulates all aspects of the proposal. This page often requires several revisions before reaching the final version that is submitted. A nice review of the approach to writing the aims section is available at https://www.niaid.nih.gov/grants-contracts/draft-specific-aims for those who are interested in learning more. A sample aims section from a successful medical education grant application has also been included as an Appendix to this paper.

The remainder of the project description section is frequently divided into three components: significance, innovation, and approach. The significance section describes the background literature and highlights the knowledge gap that will be addressed by completing the proposed project. Within medical education it is essential to include a discussion of the underlying educational theory or conceptual framework upon which the study is based.^18^,^19^ The innovation section should describe why the proposed project is novel and how this will contribute to the medical literature in the proposed area. This can include contributions to both learner education and patient care. It is also important to consider the review audience for your grant. Compositions of grant review committees vary greatly but are often primarily comprised of researchers in areas outside of medical education. Therefore, when composing the significance and innovation sections within the grant, the argument must be clear and persuasive to a reviewer unfamiliar with the current state of professional education.

Finally, the approach section details how the work will be completed. This should include details on study design, recruitment and sample size, outcome measures, analytic techniques, and consideration of the potential limitations. The clarity and technical accuracy of this section can be an important signal to a reviewer of the probability of an applicant successfully completing their proposed research program. Funders generally place a significant weight on a high likelihood of a successful return on their investment; so concerns that the analytical approach is underdeveloped or flawed can be fatal. Of special consideration for educational researchers is the description of methods less common in clinical or bench research; for example, qualitative research methodology may encounter a negative bias from quantitatively focused scholars.

In general, reviewers expect to be able to understand your project without having to read substantially about your methods elsewhere. Including appropriate references is mandatory but making reviewers work harder than they expected to is counterproductive to your goal of achieving funding. A complete bibliography may be included either at the end of the project description section or as a separate document within the application. Once again, it is important to note that the grant reviewer may not be familiar with the specific topic or may be from a different specialty or field. Therefore, it can be valuable to have a non-physician review the application to ensure that it is easily understood.

### Biographical Sketches

Biographical sketches, or biosketches, are often required for all key personnel within the investigative team. These differ in format from traditional curriculum vitaes, especially in that there generally is an expectation that they will be formatted per the National Institutes of Health (NIH) requirements. Biosketches consist of the investigator’s professional positions and educational history along with their respective publications that are most relevant to the field of medical education. These publications often include a short description of how they have impacted the relevant field of research. These may also include publications demonstrating the investigator’s experience with the specific methodology proposed for the study (e.g., qualitative, systematic reviews). Examples of well-written biosketches can be found in several locations, including on the NIH website (https://grants.nih.gov/grants/forms/biosketch.htm).

### Budget

Funding opportunities can range from hundreds to millions of dollars depending upon both the agency and the scope of the work being performed. Typically, medical education grant programs award smaller funding amounts when compared to strictly clinical or laboratory grant programs. In part, this may be due to the higher expected costs incurred with patient care and laboratory maintenance. Whatever the underlying reasoning, medical education researchers must be conscious of this difference in order to adequately budget their research resources and advocate for commensurate consideration when undergoing promotional review. Often, the funder will define the total amount of the award to be given, including the amount allowed for both direct and indirect costs.

Funding related to direct costs is paid directly to the investigative team for the study and can include investigator salaries, statistical support, supplies, simulation laboratory time, and subject recruitment. Funding for indirect costs is paid to the university or institution as overhead to cover the overall costs associated with supporting research. It is important to review the specific budget requirements, as some grants do not allow salary support or indirect costs. The budget should specifically and deliberately delineate any areas where funds are needed and how they will be used. This should be complete with the specific amounts and justification for each budgeted item to ensure that the funds are appropriately distributed. The requested amount should not exceed the maximum allowed by the funding agency.

### Facilities, Resources, and Environment

Most grant applications will request a description of the available facilities, resources, and the intellectual environment where the work associated with the project will be conducted. This should include specific details of the available resources as they pertain to the proposed project (e.g., computers, laboratory space, and office space). Specific to medical education research, one might also want to include the educational environment, including access to the learners and logistics of training. This can also include the number of learners available and prior medical education research conducted in this location. In addition to the above components, this section must also include support services available within the institution. These resources can include medical librarians to help with literature searches, a clinical translational science institute to assist with study design, or a statistician to assist with data analysis.

Finally, this section should discuss the research environment within the department and the greater institution. This may include a list of collaborators, recent publications, and other funded projects as a way to highlight the successful completion of other work. Here again, this section can be an important signal to reviewers that the proposed research project will be successfully completed. Institutions with well-documented histories of successfully completing funded research demonstrate an environment that is conducive to a successful return on the investment made by the funder. This can be especially important for more junior researchers and for educational researchers with whom grant reviewers may be less familiar with their work.

### Letters of Support

Letters of support further illustrate the level of commitment from the investigative team, the leadership of their respective departments, and the universities involved in the project. Depending upon the funding agency, letters of support may be required from key personnel involved in the project (e.g., department chairs or division chiefs, medical school deans, or other leadership within the requesting institution). These letters should clearly document the necessary resources, the commitment from the investigative team, and the institutional support to ensure success of the proposed work.

### Additional Grant Application Materials

Depending upon the type of funding mechanism sought, there are several other important documents that may be required. These may include a project timeline, description of the ethical treatment of subjects, and proof of institutional review board submission or approval. Similarly, for training awards a specific plan for career development is a necessary and vital component of the grant application process. These awards are often fundamentally different from grants that fund a specific medical education research question. Rather, training awards can fund specific professional development programs, such as a fellowship. Applicants seeking a training award must clearly delineate a specific and achievable plan for professional development, as well as how the funds will be used to achieve this plan. This is also required for certain career development (K) awards that are available through the NIH.

### Grant Evaluation

Grant applications are evaluated using multiple different methods; however, the most common scoring method is that used by the NIH. The NIH scoring tool evaluates several areas of the grant (i.e., significance, innovation, approach, investigators, environment, and overall impact), with each given a score from 1 (exceptional) to 9 (poor). The reviewer scores are averaged and multiplied by 10, resulting in an overall score.^20^ Lower numerical scores equate to a more competitive grant application. The funding range using the NIH rubric is typically 10–30, though this can depend on several factors. Funding is ultimately determined by the final score and is rated based on its congruence with the institution’s mission, available funding, and comparison to the application cohort. Given that the NIH approach is often the underlying rubric for application review, it may be beneficial to review the specific criteria and questions used in the reviews. This is especially true for educational researchers who may not have previously submitted to the NIH or similar organizations. Additional information is on this available at https://grants.nih.gov/grants/peer/guidelines_general/Review_Criteria_at_a_glance.pdf.

### Available Grant Opportunities

While there are a number of grants available for research, significantly fewer are available in medical education. One of the more significant challenges to obtaining grant funding is awareness of which grants are available. The accompanying Table provides a list of grants focusing on medical education research (Table). This includes a variety of regional, national, and international grants with website links, their missions, funding amounts, and annual submission deadlines. While most grants are annual, readers should note that some occur less frequently, while others have rolling deadlines. Researchers should also seek out local opportunities, as many institutions also have internal grant-funding opportunities. Finally, novice grant writers may not realize that funding program officers generally welcome contact prior to the application submission. Reaching out, especially when potentially coming from a non-traditional researcher background, may help improve your application and, ultimately, the chance for successful funding.

## LIMITATIONS

It is important to consider several limitations with respect to the current paper. First, this publication serves as a primer for medical education researchers interested in obtaining grant funding. While this is intended to provide an overview of the major components of grant funding, readers are advised to read *The Grant Application Writer’s Workbook* by Russell and Morrison if they are interested in learning more. Additionally, many of the recommendations are based upon the authors’ combined experience, as there is limited empirical data on effective grant-writing. However, the authors are experienced grant writers, having received over $4 million in grant funding. Finally, the grant list in the Table includes the majority of medical education grant funding opportunities. However, it is possible that there are additional medical education grant opportunities that are not included in the Table.

## CONCLUSION

Obtaining grant funding is a fundamental component of a successful research career, but it can also be challenging, especially in the field of medical education. Successful applications must meet specific structural requirements. This paper provides an overview of grant opportunities within medical education and strategies for successful grant applications. After reading this paper, researchers should feel more knowledgeable and confident with applying for medical education research grants.

## Supplementary Material



## Figures and Tables

**Table:** Available medical education grant opportunities.

Name of Grant	Website	Mission	Funding amount	Submission deadline
AMEE Seed Grants	https://amee.org/awards-prizes/research-grant-award-programme	To promote scholarship in healthcare professions education to advance knowledge and best practices in education as well as to build a community of scholars working in the field.	£10,000	February
AstraZeneca Medical Education Research Grants	https://www.astrazenecagrants.com/home.html	To support quality independent medical and scientific education and sponsorships that enhances patient care.	Variable	Varies by grant
Bristol-Myers Squibb Independent Medical Education Grants	https://www.bms.com/about-us/responsibility/IME.html	To support innovative, high quality medical education that closes gaps in health care professional knowledge, strengthens their professional competence, and improves patient health outcomes.	Not listed	Not listed
NBME Stemmler Grant	http://www.nbme.org/research/stemmler.html	To support research or development of innovative assessment approaches that will enhance the evaluation of those preparing to, or continuing to, practice medicine.	$150,000	July* January**
SACME Research Grant	https://sacme.org/SACME_Grants/	To promote the highest value in patient care and health of the public through the scholarship of continuing medical and interprofessional education.	$50,000	December* March**
SDRME Synthesis Paper Grant	http://sdrme.org/scholarship.asp	To support the writing of review/synthesis papers that make a substantial contribution to advancing practice, theory, or research in medical education.	$4,000	September
Spencer Small Research Grants	https://www.spencer.org/small-research-grants	To support academic work that will contribute to the improvement of education, broadly conceived.	$50,000	February, May, August, November
Lyle Spencer Research Award	https://www.spencer.org/lyle-spencer-research-awards	To support intellectually ambitious research oriented to improving the practice of education, independent of any particular reform agendas or methodological strictures.	$1,000,000	October
Teleflex Medical Education Grants	https://www.teleflex.com/usa/about-us/grants/medical-educational-grants/	To support genuine medical education that meets defined clinical educational needs.	Not listed	No due date requirement
The Alfred P. Sloan Foundation Higher Education Grant	https://sloan.org/grants/apply	To support original research and education related to science, technology, engineering, mathematics, and economics.	Not listed	Not listed
AAMC MESRE Grant	https://www.aamc.org/members/gea/gea_sections/mesre/	To enhance the quality of research in medical education and to promote its application to educational practice.	$20,000	February* May**
AHRQ Grants	https://www.ahrq.gov/funding/process/index.html	To produce evidence to make health care safer, higher quality, more accessible, equitable, and affordable.	Variable	Varies by grant
AMA Foundation Grant	https://www.ama-assn.org/about/community-health-programs	To improve the health of all Americans by supporting community health and medical education programs.	$40,000 – $60,000	December
CORD EMF Grants	https://www.cordem.org/opportunities/cord-grants/	To provide a vehicle for emergency medicine education researchers early in their career that promotes the development of well-conceived projects while allowing for grant writing experience and recognition of successful grant applications.	$10,000 $25,000	February
Gold Foundation Picker Gold Challenge Grant	http://www.gold-foundation.org/programs/picker-gold-challenge-grants-for-residency-training/	To support the research and development of successful patient-centered care initiatives and best practices in the education of our country’s future physicians.	$15,000 – $25,000	March* May**
Gold Foundation Mapping the Landscape Grant	http://www.gold-foundation.org/programs/research/mtl/	To promote widespread understanding of the state of research on humanism in healthcare; catalyze further research in this area; and promote the integration of humanistic principles into health professions education, clinical learning environments, accreditation standards and healthcare policy.	$5,000	June
Hearst Foundations Grant	https://www.hearstfdn.org/applying-reporting/how-to-apply/	To fund educational institutions demonstrating uncommon success in preparing students to thrive in a global society with a focus on higher education.	$50,000	Not listed
SAEM Education Project Grant	http://www.saem.org/saem-foundation/grants/funding-opportunities/what-we-fund/educational-research-grant	To foster innovation in teaching, education, and educational research in emergency medicine for faculty-, fellow-, resident- and medical student-level learners.	$20,000	August
The Josiah Macy, Jr. Foundation Grants	http://macyfoundation.org/apply	To support research in interprofessional education and teamwork, new curriculum content, new models for clinical education, career development in health professions education, and education for the care of underserved populations.	Not listed	No due date requirement
USDOE FIPSE Grant	https://www2.ed.gov/about/offices/list/ope/fipse/index.html	To spur the development of innovations that improve educational outcomes, makes college more affordable for students and families, and develops an evidence base of effective practices.	Variable	June
AAMC GEA Regional Grants	https://www.aamc.org/members/gea/regions	To advance medical education and medical educators through faculty development, curriculum development, educational research, and assessment in undergraduate, graduate, and continuing medical education.	$3,000 – $7,000	Varies by region

*AMEE*, Association for Medical Education in Europe; *NBME*, National Board of Medical Examiners; *SACME*, Society for Academic Continuing Medical Education; *SDRME*, Society of Directors of Research in Medical Education; *AAMC*, Association of American Medical Colleges; *MESRE*, Medical Education Scholarship Research and Evaluation; *AHRQ*, Agency for Healthcare Research and Quality; *AMA*, American Medical Association; *CORD*, Council of Emergency Medicine Residency Directors; *EMF*, Emergency Medicine Foundation.

* Due date for letter of intent.

** Due date for full proposal.

*SAEM*, Society for Academic Emergency Medicine; *USDOE*, United States Department of Education; *FIPSE*, Fund for the Improvement of Post-secondary Education; *GEA*, Group on Educational Affairs.
