# Supplementary material for: Show Me the Money: Successfully Obtaining Grant Funding in Medical Education
Source: West J Emerg Med. 2018 Nov 19;20(1):71–7. doi: 10.5811/westjem.2018.10.41269 (PMC6324695; doi:10.5811/westjem.2018.10.41269)
Supplement: Supplementary file 1 [file wjem-20-71-s001.docx]

**Appendix.** Sample Aims Page.

The proposed project will include three aims.

**AIM #1:**

To develop and test a predictive model of persistent interest in Emergency Medicine into actual practice.

**Hypothesis:** While accounting for the previously established importance of lifestyle and income in medical student career choice, additional factors such as academic ability, gender, and underrepresented minority status (URM) will also be significant predictive factors in persistence in an interest in Emergency Medicine.

**AIM #2:**

To identify and quantify barriers to entering Emergency Medicine that are not based on intellectual ability or student choice in order to provide evidence for future policy intervention.

**Hypothesis:** Decreased representation of women and URM physicians in Emergency Medicine is multifactorial (including unintended discouragement to enter EM) and is not just a result of differences in the previously described lifestyle and income preferences.

**AIM #3:**

To create and distribute an evidence-based predictive tool (Counseling Dashboard) for use by medical educators. This Counseling Dashboard would provide probabilities of persistent career interest in Emergency Medicine and potentially allow for early intervention and support for students who might choose a non-EM career based on factors such as gender or race. This program would be simple enough in use for national distribution to clerkship directors, residency program directors, and other researchers.

**Hypothesis:** The effectiveness of career counseling will be improved for medical students considering a career in Emergency Medicine by use of an evidence-based computer predictive tool. Such a tool could help increase representation of students who would be interested in Emergency Medicine but are not choosing this field.

**IMPACT:**

This project will have an *immediate impact* on changing the conversation on career selection in Emergency Medicine (EM). It additionally will provide medical school clerkship directors and residency program directors an evidence-based assessment of students that may benefit from additional outreach and support as they consider EM. More importantly, this project can have *wide-spread, long-term impact* on considerations of physician representation in Emergency Medicine by providing a much more comprehensive analysis of issues in the pipeline and recruitment to the field.
